# Endoscopic ultrasound-guided jejunoduodenostomy followed by biliary stenting using an ultrathin endoscope

**DOI:** 10.1055/a-2590-2500

**Published:** 2025-05-19

**Authors:** Koichiro Mandai, Takato Inoue

**Affiliations:** 1 Department of Gastroenterology, Kyoto Second Red Cross Hospital, Kyoto, Japan

Endoscopic biliary drainage is challenging in patients with left hepatectomy (LH) and extrahepatic bile duct (EHBD) resection, as balloon enteroscope-assisted endoscopic retrograde cholangiopancreatography (BE-ERCP) may not always be feasible, and endoscopic ultrasound (EUS)-guided hepaticogastrostomy is not an option. We describe a case of EUS-guided jejunoduodenostomy (EUS-JDS) followed by biliary stenting in such a patient ( Video 1 ).

Endoscopic ultrasound-guided jejunoduodenostomy followed by biliary stenting using an ultrathin endoscope through an endosonographically created route.Video 1

An 82-year-old woman, with LH and EHBD resection for cholangiocarcinoma, developed obstructive jaundice following cancer recurrence near the hepaticojejunostomy anastomosis (HJA). BE-ERCP failed, and we attempted EUS-JDS for biliary access by puncturing the jejunum near the HJA using a 19G needle ( Fig. 1 **a** ). Although the intrahepatic bile duct (IHBD) was visualized with contrast injection from the jejunum, contrast drainage was poor, suggesting a stricture near the HJA. After inserting two guidewires into the jejunum using a double-lumen cannula, the tract was dilated with a 4-mm balloon ( Fig. 1 **b** ). A fully covered metal stent (10 mm × 7 cm) was placed from the jejunum to the duodenum; a 7-Fr double-pigtail plastic stent (10 cm) was inserted through the metal stent to prevent migration 1 ( Fig. 1 **c** ).

**Fig. 1:**
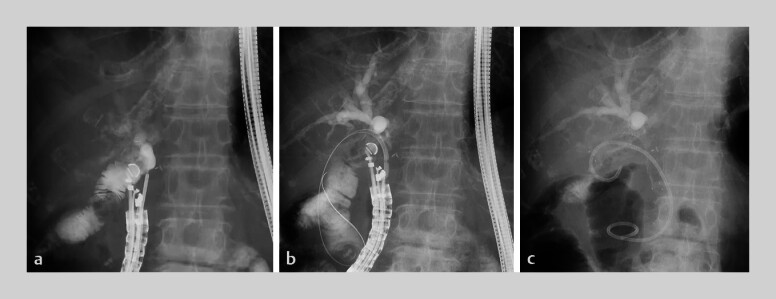
**a** The jejunum near the hepaticojejunostomy anastomosis punctured using a 19G needle. **b** The puncture route is dilated with a 4-mm balloon. **c** Endoscopic ultrasound-guided jejunoduodenostomy performed using a 10-mm fully covered metal stent combined with a 7-Fr double-pigtail plastic stent.

Eight days later, the EUS-JDS stents were removed, and an ultrathin endoscope (GIF-1200N; Olympus,) was advanced into the jejunum via the EUS-JDS route ( Fig. 2 **a** ). The guidewire was inserted into the right IHBD through the HJA stricture ( Fig. 2 **b** ). An 8-mm uncovered metal stent with a 5.4-Fr delivery system (YABUSAME Neo; Kaneka Medical) was placed from B5 to the jejunum, followed by a second one from B8 to the EUS-JDS route 2 ( Fig. 2 **c** ). The patient’s jaundice improved without any adverse events.

**Fig. 2:**
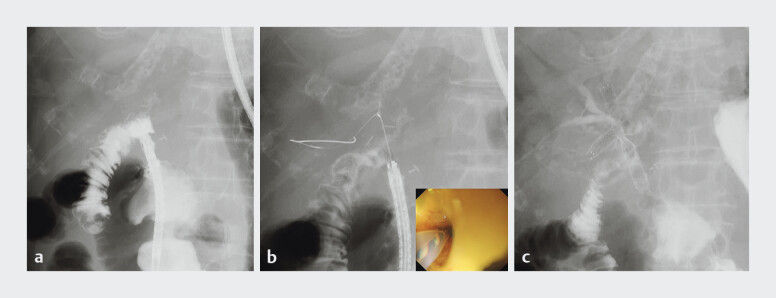
**a** After the stents placed during endoscopic ultrasound-guided jejunoduodenostomy were removed, no contrast leakage is observed. **b** A guidewire inserted into the right intrahepatic bile duct using an ultrathin endoscope. **c** An 8-mm uncovered metal stent with a 5.4-Fr delivery system is placed from B5 to the jejunum, followed by a second stent through the stent mesh from B8 to the endoscopic ultrasound-guided jejunoduodenostomy route.

A lumen-apposing metal stent is useful for EUS-guided digestive tract anastomosis but unavailable in some countries. Our technique provides a viable alternative for biliary drainage in patients with LH and EHBD resection.

Endoscopy_UCTN_Code_TTT_1AS_2AH

## Notes

Endoscopy E-Videos https://eref.thieme.de/e-videos
*E-Videos* is an open access online section of the journal *Endoscopy* , reporting on interesting cases and new techniques in gastroenterological endoscopy. All papers include a high-quality video and are published with a Creative Commons CC-BY license. Endoscopy E-Videos qualify for HINARI discounts and waivers and eligibility is automatically checked during the submission process. We grant 100% waivers to articles whose corresponding authors are based in Group A countries and 50% waivers to those who are based in Group B countries as classified by Research4Life (see: https://www.research4life.org/access/eligibility/ ). This section has its own submission website at https://mc.manuscriptcentral.com/e-videos .
